# *S. aureus* MscL Is a Pentamer In Vivo but of Variable Stoichiometries In Vitro: Implications for Detergent-Solubilized Membrane Proteins

**DOI:** 10.1371/journal.pbio.1000555

**Published:** 2010-12-07

**Authors:** Michael R. Dorwart, Robin Wray, Chad A. Brautigam, Youxing Jiang, Paul Blount

**Affiliations:** 1 Department of Physiology, University of Texas Southwestern Medical Center at Dallas, Dallas, Texas, United States of America; 2 Howard Hughes Medical Institute, University of Texas Southwestern Medical Center at Dallas, Dallas, Texas, United States of America; 3 Department of Biochemistry, University of Texas Southwestern Medical Center at Dallas, Dallas, Texas, United States of America; Brandeis University, United States of America

## Abstract

While the bacterial mechanosensitive channel of large conductance (MscL) is the best studied biological mechanosensor and serves as a paradigm for how a protein can sense and respond to membrane tension, the simple matter of its oligomeric state has led to debate, with models ranging from tetramers to hexamers. Indeed, two different oligomeric states of the bacterial mechanosensitive channel MscL have been resolved by X-ray crystallography: The *M. tuberculosis* channel (MtMscL) is a pentamer, while the *S. aureus* protein (SaMscL) forms a tetramer. Because several studies suggest that, like MtMscL, the *E. coli* MscL (EcoMscL) is a pentamer, we re-investigated the oligomeric state of SaMscL. To determine the structural organization of MscL in the cell membrane we developed a disulfide-trapping approach. Surprisingly, we found that virtually all SaMscL channels in vivo are pentameric, indicating this as the physiologically relevant and functional oligomeric state. Complementing our in vivo results, we purified SaMscL and assessed its oligomeric state using three independent approaches (sedimentation equilibrium centrifugation, crosslinking, and light scattering) and established that SaMscL is a pentamer when solubilized in Triton X-100 and C_8_E_5_ detergents. However, performing similar experiments on SaMscL solubilized in LDAO, the detergent used in the crystallographic study, confirmed the tetrameric oligomerization resolved by X-ray crystallography. We further demonstrate that this stoichiometric shift is reversible by conventional detergent exchange experiments. Our results firmly establish the pentameric organization of SaMscL in vivo. Furthermore they demonstrate that detergents can alter the subunit stoichiometry of membrane protein complexes in vitro; thus, in vivo assays are necessary to firmly establish a membrane protein's true functionally relevant oligomeric state.

## Abstract 2

Detergent-induced rearrangements of membrane-protein subunits explain why two MscL channel stoichiometries have been resolved by X-ray crystallography - but *S. aureus* MscL is truly a pentamer in vivo.

## Author Summary

The ability to detect mechanical forces is at the basis of not only the senses of touch hearing and balance but also cardiovascular and osmotic regulation. One of the primary ways that organisms detect forces is through mechanosensitive channels, and mechanosensation is so vital that essentially all organisms have at least one such sensor. Indeed, the best-studied mechanosensitive channel is from bacteria, and because relatively little is known of mechanosensors from higher organisms, these channels are a model for how a protein can sense and respond to mechanical forces. Although the bacterial mechanosensitive channel MscL has been well studied, the simple issue of how many subunits it has is hotly debated. There are even two published crystal structures showing either tetrameric or pentameric complexes. Here we show that the channel is actually pentameric in vivo and that the detergent used to solubilize the protein can rearrange the complexes from pentamers to tetramers. The finding that detergents can have such a profound effect on structure may have broad implications for the study of other membrane proteins.

## Introduction

The bacterial mechanosensitive channel MscL serves as a biological “emergency release valve,” allowing rapid loss of solutes in response to a sudden decrease in the osmolarity of a bacterium's environment [1]. It is perhaps the best characterized mechanosensor [2], thus serving as a paradigm of how a membrane protein can detect and respond to mechanical forces [3]. Ironically, something as simple as the stoichiometry of the MscL complex has plagued the field with debate since its inception.

The original model for the *E. coli* MscL (EcoMscL) stoichiometry was a homo-hexameric organization, which was suggested by crosslinking and the study of tandem subunits [4]. This model then appeared to be supported by low-resolution two-dimensional crystallization of EcoMscL [5]. But the subsequent elucidation of the *M. tuberculosis* channel (MtMscL) by X-ray crystallography [6] then suggested a pentameric organization, at least for this orthologue. This result led to a re-evaluation of EcoMscL stoichiometry [6],[7], which supported a pentameric organization and brought into question whether the two-dimensional crystallization data could be fit by 5-fold as well as 6-fold symmetry. Thus, the field transiently seemed to have settled that MscL was most likely a pentamer. However, the recent crystallographic structure of the *S. aureus* homolog (SaMscL) reveals a tetramer variant [8]. This latter finding has again raised questions regarding the true oligomeric state of MscL and evokes the possibilities either that MscL from different species assemble into complexes with different stoichiometries or that the channel exists as multiple functional oligomeric complexes in the cell membrane. Therefore, we set out to identify the MscL oligomeric state in the cell membrane and to understand how the SaMscL channel, which shares approximately 40% sequence identity with EcoMscL and MtMscL [8], could exist in the non-pentameric subunit organization resolved by X-ray crystallography. We found not only that the true in vivo oligomeric state of SaMscL is a pentamer but also that at least one detergent, LDAO, artificially but reversibly reorganizes this structure into a tetrameric stoichiometry.

## Results

**Figure 1:**
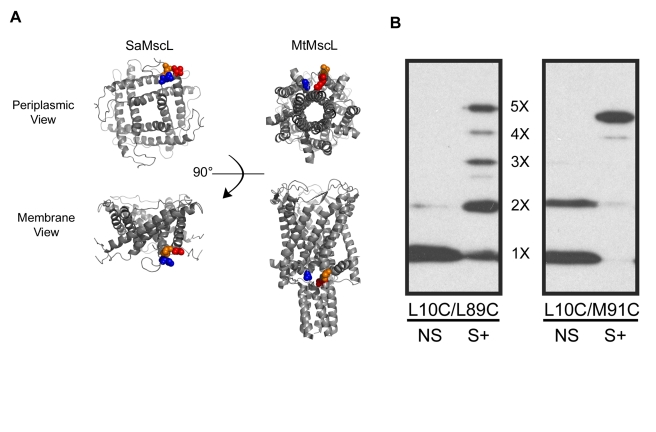
*S. aureus* MscL is a pentamer in the cell membrane. (A) Two orientations of the SaMscL and MtMscL crystal structures are shown with the molecules viewed from the periplasmic side (top) and from the lipid bilayer (bottom). The locations of the residues used to perform the in vivo disulfide crosslinking experiments are highlighted on the structures in surface representation: blue represents L10 (SaMscL) and A10 (MtMscL), orange represents T89 (SaMscL) and T96 (MtMscL), and red represents M91 (SaMscL) and R98 (MtMscL). (B) Western blot analysis of L10C/L89C and L10C/M91C SaMscL channels after in vivo disulfide bond trapping using copper-phenanthroline in both osmotically shocked (S+) and non-shocked (NS) samples.

With multiple oligomeric states identified in vitro, we devised a disulfide-trapping strategy to identify the oligomeric state of MscL in bacterial membranes. Using this approach, we were able to directly measure the subunit stoichiometry of SaMscL in vivo by generating a series of double-cysteine mutants in regions predicted to be in close proximity from both existing crystal structures (Figure 1A). These modifications allow crosslinking in the cell membrane via disulfide bonds as previously described [9],[10]. Briefly, the cells were osmotically shocked in the presence of the oxidizing agent copper-phenanthroline, centrifuged, and then immediately resuspended in SDS loading buffer to avoid unnecessary manipulation of the proteins. As we previously observed [9], osmotic shock increased disulfide bridging, presumably because reductants including glutathione and thioredoxin are released from the cell [11], thus decreasing the reducing potential of the cytosol. The protein content of the lysed cells was evaluated using SDS-PAGE and molecular weight standards. For the L10C/L89C or L10C/M91C SaMscL channels, we were able to observe efficient in vivo disulfide trapping (Figure 1B). For the L10C/L89C protein, a range of oligomeric states was observed, a pentamer being the maximal state. However, for the L10C/M91C protein, one primary band was observed that reflects a pentameric complex. These data demonstrate that the pentamer is by far the major species within the cell membrane and thus appears to be the true structural and functional unit in vivo.

**Figure 2:**
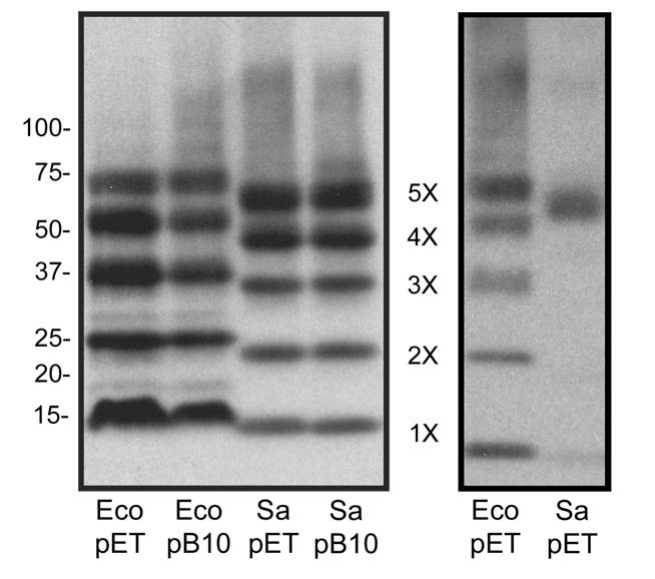
*E. coli* and *S. aureus* MscL are pentameric multimers by crosslinking. Western blot analysis of DSS-treated *E. coli*-MscL (Eco) and *S. aureus*-MscL (Sa) expressed with either the pET21a (pET) or pB10 vector show five distinct bands at both expression levels. Molecular weight markers are shown to the left of the blot and the approximate location of monomers (1×) to pentamers (5×) is shown between the blots. The right-hand blot shows a separate experiment where the majority of the EcoMscL and the SaMscL protein are in the pentameric and monomeric forms.

**Figure 3:**
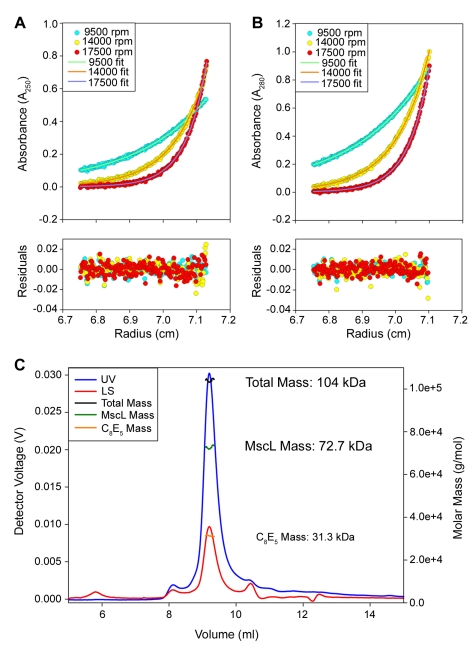
Purified *S. aureus* MscL is pentameric by equilibrium sedimentation centrifugation and SEC-MALS. Sedimentation equilibrium centrifugation was performed on 40 µM SaMscL in the neutrally buoyant detergent C_8_E_5_. The upper two panels in (A) and (B) show scans from three different rotor speeds monitored at either 250 nm (A) or 280 nm (B). The lines represent fits to the data of a single species model that yield a calculated protein mass of 71.2 kDa with a local rmsd of 0.005799 (A) and 0.005059 (B) (SaMscL monomer mass is 14.4 kDa; pentamer mass is 72.2 kDa). The lower two panels in (A) and (B) show the residuals from the data fitting. A representative SEC-MALS experiment of SaMscL in C_8_E_5_ is shown in (C) with the UV chromatogram colored blue and the 90° light scattering chromatogram colored red. The total computed mass is shown as the black line across the elution peak, with the SaMscL and C_8_E_5_ mass shown in green and orange, respectively.

With SaMscL pentamers being the predominant oligomeric state in the cell membrane, we wanted to understand why SaMscL crystallized as a tetramer. We began by confirming the previously observed pentameric and tetrameric states of MscL by performing crosslinking experiments of purified EcoMscL and SaMscL, visualizing their oligomeric state by Western blot analysis (Figure 2). Five crosslinked EcoMscL products were observed, consistent with previous reports showing that EcoMscL is a pentamer [6],[7]. In contrast with previous findings [8], SaMscL also presented five crosslinked products, suggesting that SaMscL also exists in a pentameric oligomeric state. No difference was observed between proteins expressed by a high-expressing plasmid (pET) and those encoded a moderate-expressing plasmid (pB10), demonstrating that expressions levels have no effect on MscL oligomerization. To substantiate these results, sedimentation equilibrium analytical ultracentrifugation (SE) was performed to directly measure the mass of the SaMscL channel. The results for SaMscL were fit to a single-species model and yielded a molar mass of 71.2 kDa, consistent with a pentameric subunit organization (SaMscL theoretical pentameric molar mass is 72.2 kDa) (Figure 3A, B). As a third independent method to directly measure the mass of the SaMscL channel, we performed size exclusion chromatography multi-angle light scattering (SEC-MALS) experiments [12]. Analysis of nine independent SEC-MALS experiments resulted in an average calculated SaMscL mass of 72.8±0.8 kDa (Ave ± SD, *n* = 9), again consistent with a pentameric subunit organization of SaMscL (representative experiment shown in Figure 3C).

The data presented in Figures 2 and 3 show three independent approaches that all confirm the pentameric oligomeric state of SaMscL, in agreement with our in vivo crosslinking results. These data are also consistent with the MtMscL crystal structure [6],[13] and biochemical studies of EcoMscL [7] but inconsistent with the subunit organization in the SaMscL crystal structure [8]. The major difference in the protein handling between our experiments and those previously published for the SaMscL channel [8] was the choice of solubilizing detergent. Our measurements were made in the presence of either Triton X-100 (crosslinking) or pentaethylene glycol monooctyl ether (C_8_E_5_) detergent (SE and SEC-MALS), not Lauryldimethylamine-oxide (LDAO), the detergent used previously to crystallize SaMscL and evaluate its oligomeric state [8]. To determine if the solubilizing detergent could account for the discrepancies in the data, we tested whether LDAO may affect the oligomeric state of SaMscL.

**Figure 4:**
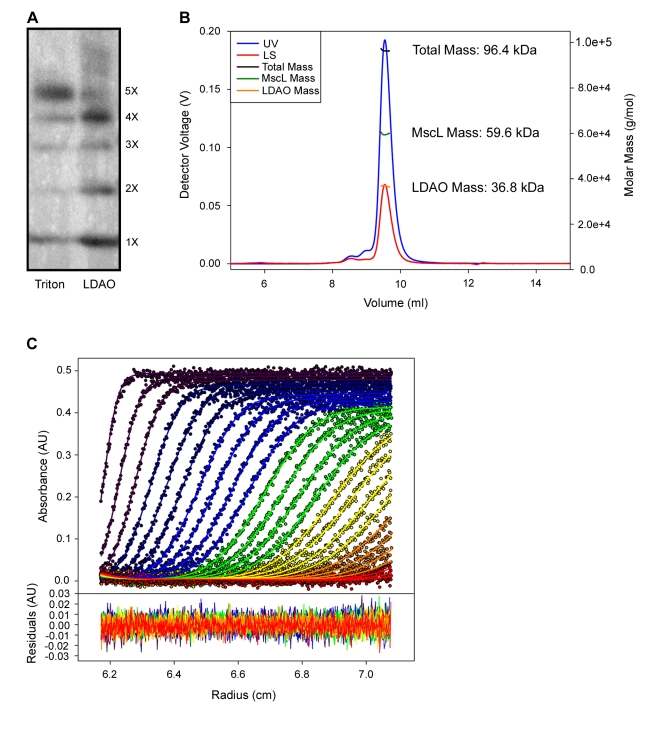
LDAO stabilizes a MscL tetrameric oligomeric state as measured by crosslinking, SEC-MALS, and Sedimentation Velocity Centrifugation. (A) Western blot analysis of DSS-treated SaMscL solubilized with either Triton-X100 or LDAO yield different quantities of tetramers and pentamers. (B) A representative SEC-MALS experiment of SaMscL in LDAO is shown with the UV chromatogram colored blue and the 90° light scattering chromatogram colored red. The total, SaMscL, and LDAO masses are shown as black, green, and orange lines across the elution peak, respectively. The mass of the SaMscL monomer is 14.4 kDa and the tetramer mass is 57.8 kDa. (C) SaMscL in 4 mM LDAO was subjected to sedimentation velocity centrifugation at 50,000 rpm, and the data were analyzed using the noninteracting “species model” of SEDPHAT. Four species, including the detergent micelles, were analyzed. The molar mass of the dominant sedimenting species was 62.5 kDa. In the *upper part*, the individual data points are depicted as circles, and the best-fit model to those data is shown as lines. The data and fit lines are color coded by color: Violet for the earliest scans, then progressing through indigo, blue, green, yellow, orange, and red as the scans go further forward in time. For clarity only every other scan used in the data analysis is shown. In the *lower part*, the residuals between the data points and the fitted line are shown and color coded as above.

We performed SaMscL crosslinking experiments in the presence of LDAO and found essentially no crosslinked pentamer and a substantial increase in tetramers when compared to the experiments performed in Triton X-100 (Figure 4A). To confirm that LDAO was altering the oligomeric state of the SaMscL channel rather than simply affecting our crosslinking efficiency, we directly measured the mass of the SaMscL protein in the presence of LDAO by SEC-MALS (representative experiment shown in Figure 4B). The results show that the average protein mass in the presence of LDAO is 60.0±2.8 kDa (Ave ± SD, *n* = 8), a mass that is dramatically reduced compared to the C_8_E_5_ experiments (72.8±0.8 kDa) and that is consistent with a tetrameric oligomeric state (theoretical SaMscL tetramer is 57.8 kDa). As a third independent approach to assess the oligomeric state of the protein, we performed sedimentation velocity experiments on the SaMscL protein in LDAO. Two different methods were used to calculate the molar mass of the protein from these data (see Methods for details). Both resulted in similar values: 62.3±1.3 kDa (“*s* and *D*” method, Figure 4C) and 64±3 kDa (“Stokes radius” method) (Weighted Ave ± 1σ). These results again show a protein molar mass well below that of a pentamer, consistent with LDAO affecting the oligomeric state of the SaMscL channel.

**Figure 5:**
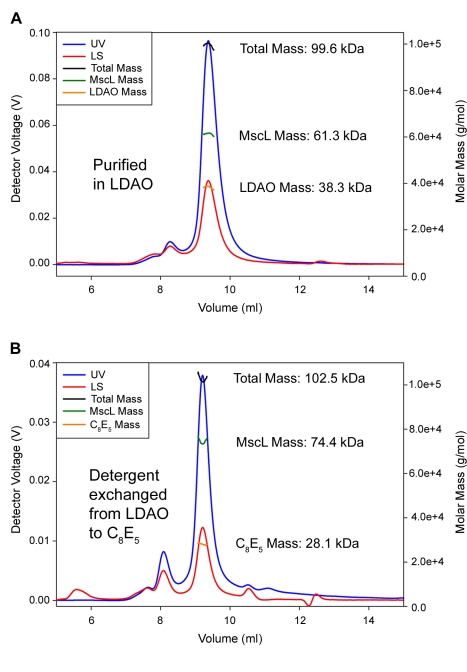
The *S. aureus* MscL stoichiometry change is reversible. (A) The oligomeric state of SaMscL in 4 mM LDAO was determined using SEC-MALS, which measured a protein mass of 61.3 kDa, consistent with a tetrameric channel (top chromatogram). (B) The SaMscL-LDAO sample was exchanged into C_8_E_5_, and the following day the oligomeric state of SaMscL-C_8_E_5_ sample was measured by SEC-MALS. Analysis of the SEC-MALS results for the SaMscL-C_8_E_5_ sample showed a protein mass of 74.4 kDa, consistent with the channel in a pentameric state.

We also tested the reversibility of this oligomeric state reorganization by using SaMscL purified in LDAO and then exchanged the LDAO detergent for C_8_E_5_. This protocol resulted in the SaMscL mass increasing from 61.4±0.7 kDa (Ave ± SD, *n* = 3) to 72.0±2.6 kDa (Ave ± SD, *n* = 4) as measured by SEC-MALS (Figure 5). The choice of initial detergent made no difference in the reversibility, as purifying SaMscL in C_8_E_5_ and then exchanging the detergent for LDAO showed a mass decrease from 72.2±0.5 kDa in C_8_E_5_ (Ave ± SD, *n* = 5) to 58.7±0.7 kDa in LDAO (Ave ± SD, *n* = 3). Hence, the oligomeric rearrangement is reversible and is completely dependent upon the detergent solubilizing the MscL protein.

## Discussion

Essentially all previous structural studies on MscL, as with most membrane proteins, have been performed on channels that have been solubilized, under the assumption that detergents do not alter the subunit stoichiometry. Our data examining the effect of different detergents on the oligomeric state of SaMscL have far-reaching implications for membrane protein research, as we now clearly demonstrate that changes in oligomeric state can and do occur upon detergent solubilization, and these alterations are not easily detected by SEC (see Figures 3C and 4B). To circumvent these artifacts associated with solubilization, we have developed an in vivo cross-linking assay. Although the pentamer is by far the major product observed in Figure 1B, very small amounts of smaller oligomers can be resolved upon overloading of protein gels and long Western blot exposures. The small amount of tetramers and smaller complexes observed upon long exposure could be incompletely assembled protein or may simply be due to the lack of disulfide bridging, which is rarely observed to be this efficient (e.g. see [9],[10]). In addition, we cannot completely rule out the possibility that oligomerization is a dynamic and even reversible process in vivo. Regardless, it seems unlikely that oligomeric species smaller than pentamers have a physiological role given that there are tens of channels per cell [14], and the smaller species compose only a few percent of the total channels. Our data definitively show that the vast majority of the SaMscL channel adopts a pentameric structure in the cell membrane, consistent with the MtMscL crystal structure [6], but incompatible with the SaMscL crystal structure [8].

A close inspection of the recent tetrameric SaMscL structure, which was speculated to be in an expanded gating intermediate state, reveals structural interactions between residues in TM1 and TM2 residues of a neighboring subunit similar to those in the closed MtMscL structure. This result was surprising because several studies using various techniques have suggested TM1 undergoes a significant clockwise rotation (as viewed from the periplasmic side) upon channel gating. The approaches supporting this interpretation include: spin labeling combined with electron paramagnetic resonance (EPR) studies [15], accessibility of sulfhydryl reagents to cysteine mutants in TM1 upon gating [16],[17], accessibility of heavy metals to engineered binding sites in the pore [18], suppression mutagenesis demonstrating interactions between TM1 and TM2 upon gating [19], and confirmation of multiple interaction sites between TM1 and TM2 upon gating by using an electrostatic repulsion approach as well as disulfide trapping [10]. Both the EPR studies as well as the heavy-metal binding in the pore strongly suggest that this rotation occurs quite early in the gating process, before ion permeation. By this model, V21 and G24 (V23 and G26 in EcoMscL) should rotate away from the pore constriction, allowing V22 and the positively charged K29 (I24 and K31 in EcoMscL) to line the open pore. The observation that this rearrangement has not occurred strongly suggests that the crystal SaMscL structure actually reflects a strained closed state due to improper oligomeriazation, rather than an expanded intermediate state.

The observed stoichiometry changes in detergents also evoke questions regarding how membrane protein subunits assemble into physiological multimeric complexes. A previous report on EcoMscL demonstrated that when the protein is translated in vitro in the absence of any lipid or detergent and is subsequently purified in the presence of Triton X-100, the channel spontaneously assembles into pentamers [20]. A separate study demonstrated that even synthetically synthesized EcoMscL will oligomerize into functional channels when incorporated into lipid membranes [21]. These studies demonstrate that the EcoMscL channel has the ability to self-assemble into the functional oligomer in vitro. Similarly, it appears that C_8_E_5_ and Triton X-100 allow SaMscL to properly fold and assemble into a pentameric structure, which correlates with the observed oligomeric state in vivo. In contrast, LDAO reorganizes SaMscL into tetramers that do not appear to exist in significant quantities in vivo, suggesting that they are not physiologically relevant. The observed self-assembly properties of EcoMscL under many conditions, combined with the reversible nature of the detergent-induced oligomeric state changes for SaMscL, may explain why Liu et al. observed channel function for the LDAO solubilized SaMscL tetramers [8]; presumably, once the tetrameric LDAO-solubilized SaMscL is reconstituted into lipids, it rearranges back into functional pentameric structures.

The crystal structure of the SaMscL was derived from a truncation mutant in which several amino acids at the C-terminus had been removed [8]. One previous study suggested that at least some C-terminus deletions of EcoMscL could lead to the formation of larger aggregates when translated in a cell-free system [22]. While we cannot rule out the possibility that the SaMscL deletion contributed to the stabilization of the tetramer in the crystal, our data do demonstrate that detergents alone can stabilize alternative oligomeric states of the full-length SaMscL in solution.

Our observations make it tempting to speculate that larger, more hydrated head groups (i.e. the head groups of C_8_E_5_ and Triton X-100) promote pentamer formation, while smaller head groups (i.e. LDAO) promote tetramer formation. However, with such a small number of detergents tested thus far, additional experiments will be required to identify what chemical properties of the detergents cause the oligomeric state change and what effect, if any, lipids may have on the stabilization of specific oligomeric states.

In sum, our data demonstrate that the physiologically active SaMscL oligomer in vivo is a pentamer and that purification in detergents can cause a reorganization of the SaMscL channel's oligomeric state. Our findings also show that SEC alone is insufficient to evaluate the usefulness of a particular detergent for the study of membrane proteins; ideally, SEC-MALS and/or analytical ultracentrifugation should be correlated with in vivo experiments that can more confidently determine the functional and physiologically relevant oligomeric state of membrane proteins.

## Materials and Methods

### Purification

SaMscL (accession number: CAG43065) was expressed using the pET21a vector in PB116 *E. coli* cells grown to O.D._600_ 0.6–0.8 and induced with 1 mM IPTG for 2–3 h at 37°C. Cells were harvested and stored at −80°C until needed. Cells containing SaMscL were lysed in 50 mM NaPi pH 8.0, 300 mM NaCl, 0.5 µg/ml DNAse, 1 mg/ml lysozyme, and 150 µl protease inhibitors (Sigma) using a French press at 16K PSI at 4°C. The cell lysate was incubated with 40 mM n-Decyl-β-D-Maltopyranoside (DM) for 1 h at 4°C and then centrifuged at 24,000× g for 30 min at 4°C to clarify the lysate. 4 mM imidazol was added to the resulting supernatant and then bound to 4 ml of Ni-NTA slury pre-equilibrated with 50 mM NaPi pH 8.0, 300 mM NaCl, 5 mM imidazol, and 40 mM DM for 1 h at 4°C. Standard metal affinity chromatography was performed with 10 column volumes of wash buffer containing 50 mM NaPi pH 8.0, 300 mM NaCl, 30 mM imidazol, 4 mM DM, and SaMscL was eluted with 50 mM NaPi pH 8.0, 300 mM NaCl, 300 mM imidazol, and 4 mM DM. The purest protein fractions were combined and concentrated using a 10,000 molecular weight cutoff filter (Amicon). Detergent exchange was accomplished by adding 40 mM Lauryldimethylamine-oxide (LDAO) or 5% (w/v) pentaethylene glycol monooctyl ether (C_8_E_5_) to the concentrated SaMscL and allowing the protein-detergent complex to equilibrate overnight at room temperature. The following day the protein was subjected to size exclusion chromatography using a superdex 200 column (GE Healthcare) in 20 mM NaPi pH 7.5, 170 mM NaCl, and 4 mM LDAO or 0.5% (w/v) C_8_E_5_. The eluting peak was collected and concentrated using a 50,000 molecular weight cutoff filter (Amicon) and used for either sedimentation equilibrium experiments or size exclusion chromatography-multiangle light scattering experiments (SEC-MALS).

### Crosslinking

SaMscL and EcoMscL were cloned into pB10 and pET21a vectors and transformed into PB104 or PB116 *E. coli* and purified as previously described [10]. 2% Triton X-100 or 44 mM LDAO was used for MscL extraction with a final concentration of 0.2% and 2.1 mM, respectively, after purification. For crosslinking reactions 5 µg of protein was incubated with 2 mM DSS (Thermo Scientific) on ice for 5 min. The reaction was quenched with a final concentration of 100 mM Tris pH 7.5 for 15 min at room temperature as recommended by the manufacturer. Samples were brought up in non-reducing sample buffer, were loaded on a Criterion 4–20% gel, and were subjected to Western as previously described [10]. The primary antibody anti-Penta His (Qiagen) was used at 1∶4,000 and the secondary Goat anti-Mouse HRP (Bio-Rad) at 1∶100,000. Blots were developed using HRP substrate (Millipore) and exposed to film.

### In Vivo Disulfide Trapping

Overnight cultures were diluted 1∶100 and grown 1 h at 37°C in LB. LB with 1 M NaCl was then added for a final concentration of 0.5 M. Cultures were then induced with 1 mM IPTG for 1 h when an OD 600 of 0.2 was reached. Cultures were either Mock shocked (0.5 M NaCl LB) or shocked (water with 1.5 µM copper phenanthroline) at a 1∶20 dilution for 15 min at 37°C. Samples were spun down at 4,000 g for 20 min and immediately re-suspended in non-reducing sample buffer, adjusted for final OD, and run on a 4%–20% gel (Bio-Rad) for Western blot analysis [10]. The primary antibody anti-Penta His (Qiagen) was used at 1∶4,000 and the secondary Goat anti-Mouse HRP (Bio-Rad) at 1∶40,000. Blots were developed using HRP substrate (Millipore) and exposed to film.

### SEC-MALS

A Shimadzu Prominence HPLC system equipped with a Shodex KW-803 SEC column was connected in line with a miniDAWN-TREOS light scattering instrument (Wyatt Technologies) and Optilab rEX refractometer (Wyatt Technologies). The light scattering and refractive index instruments were calibrated following the manufacturer's guidelines, and 40 ml of running buffer (20 mM NaPi pH 7.5, 170 mM NaCl and 4 mM LDAO or 0.5% (w/v) C_8_E_5_) was used to equilibrate the SEC column and establish stable baselines for the light scattering and refractive index instruments. 25–75 µg of purified protein was used for each SEC-MALS experiment, and data were collected using ASTRA V software (Wyatt Technologies) and processed following the manufacturer's guidelines. LDAO and C_8_E_5_ dn/dc values (0.1490 and 0.1240, respectively) were measured using the Optilab rEX following the manufacturer's guidelines and the SaMscL extinction coefficient was calculated from the amino acid sequence.

### Analytical Ultracentrifugation

Buffer density-matching experiments were performed in a Beckman Coulter XL-I Analytical Ultracentrifuge to identify the appropriate solution density to match the density of the C_8_E_5_ detergent. Volumes of 180 µL of solutions containing 20 mM NaPi pH 7.5, 0.5% (w/v) C_8_E_5_, and various concentrations of NaCl were placed in the sample sector of a dual-sectored, charcoal-filled Epon centerpiece (Beckman-Coulter; all AUC experiments were carried out in such centerpieces) that was sandwiched between sapphire windows. Identical buffers without detergent were placed in the reference sector. The centrifugation cells were centrifuged at a temperature of 25°C and a rotor speed of 50,000 rpm; an An-50Ti rotor was used for all centrifugation experiments. The formation of any detergent concentration gradient was monitored using interference optics. C_8_E_5_ was found to be neutrally buoyant in 20 mM NaPi pH 7.5, 170 mM NaCl, which was then used for all sedimentation equilibrium centrifugation experiments with 0.5% C_8_E_5_ at 25°C. 4, 8, 40, and 80 µM SaMscL samples were prepared and loaded into the assembled ultracentrifuge cells; buffer with detergent was deposited in the reference sector. Long solution columns (180 µL) were also used in this experiment. Centrifugation was performed at 25°C at 9,500, 14,000, and 17,500 rpm. The data were analyzed using SEDFIT to determine if the samples had come to equilibrium, and SEDPHAT [23] was used to fit the sedimentation equilibrium data to a single species model. Multispeed analysis with mass-conservation constraints was performed, allowing the time-invariant and radially invariant noise elements to be calculated and subtracted from the data [23]. A 68.3% confidence interval was calculated as previously described [24],[25]. The error number reported is the average of the positive and negative deviations from best mass value. For the determination of the amount of detergent bound per gram of protein (δ*_D_*), buffer without detergent was used in the reference sector, and two different concentrations (41 and 66 µM) of MscL were placed in the sample sector. The volume of each sample and reference was 390 µl. The rotor speed used was 50,000 rpm, and the temperature setting was 20°C. The data were analyzed in SEDFIT using the non-interacting discrete species model [26],[27]. The flotation of the detergent micelles (the density of LDAO is less than that of water) was explicitly accounted for by giving that species a negative sedimentation coefficient. The amounts of absorbance units and interference fringes present for the main sedimenting species were derived from this analysis. Using the calculated extinction coefficient of the protein and the known refractive index increments of the detergent and the protein, *δ_D_* was calculated as in le Maire et al. [28]. Subsequent sedimentation velocity experiments were performed with 20, 32, and 64 µM SaMscL samples in 4 mM LDAO at 50,000 rpm at 20°C. The volume centrifuged was 390 µL. In these cases, the reference buffer contained the same [LDAO] as the sample. The absorbance data (interference data were not collected) were initially analyzed using the *c*(*s*) model in SEDFIT [26]. Although LDAO has a very low extinction coefficient at 280 nm, its floatation was detectable; the range of data near to the meniscus was therefore eliminated for this analysis. In these analyses, small quantities (2%–3% of the total signal) of large species were detected. Using this information, all species, including the floating detergent, were modeled in the “species analysis” model in SEDPHAT [26]. This approach is referred to as the “*s* and *D*” method in the text, because both the sedimentation coefficient and the diffusion coefficient are refined to arrive at the buoyant molar mass (*M_b_*). *M_b_* was transformed to *M* (the molar mass of the protein) using the formula
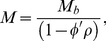
(1)where
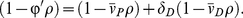
(2)
[29]

The symbol *ρ* is the solution density, 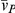 is the partial specific volume of the protein, and 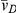 is the partial specific volume of the detergent. Any lipids bound to SaMscL were neglected in these calculations. *M_b_* was also calculated using the relationship
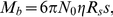
(3)where *N*_0_ is Avogadro's number, *η* is the solution viscosity, *R_s_* is the Stokes radius of the protein/detergent complex, and *s* is the sedimentation coefficient. The Stokes radius of the protein was obtained by calibrating the SEC column described above with proteins of known Stokes radius. *R_s_* for SaMscL in LDAO was 4.2 nm. Again, *M_b_* was transformed to *M* using Eq. 1. Values for 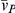, *η*, and *ρ* were estimated using the program SEDNTERP [30]. The value of 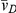 was taken as 1.13 mL/g [31]. Confidence intervals of *s* and *M_b_* were calculated as detailed above. A confidence interval for the value of Stokes radius, which was derived from a linear regression, was:
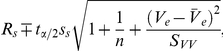
where *t* is the *t* statistic, *s_s_* is the estimate of *σ* for the regression line, *V_e_* is the elution volume of MscL from the SEC column, 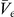 is the average elution volume of the standards, *n* is the number of standards, and *S_VV_* is the sum of squared deviations of the individual standard compared to 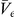. In cases of asymmetric error intervals, the *σ* was taken as the average of the difference between the boundary value and the best-fitted value. Because multiple experiments, each having different errors, were performed, the value of *M* reported in the main text is the weighted mean *μ_w_* ± the weighted *σ* (*σ*_w_), calculated using the formulas:
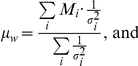


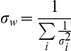
for the *I* experiments preformed (*i* = 3 in this case).

## Abbreviations

C_8_E_5_: pentaethylene glycol monooctyl ether
EcoMscL: *E. coli* MscL
LDAO: n-dodecyl-N,N-dimethylamine-N-oxide
MtMscL: *M. tuberculosis* MscL
SaMscL: *S. auresus* MscL
SDS: sodium dodecyl sulphate
SE: sedimentation equilibrium analytical ultracentrifugation
SEC-MALS: size exclusion chromatography multi-angle light scattering
